# Effectiveness of isometric exercises on disability and pain of cervical spondylosis: a randomized controlled trial

**DOI:** 10.1186/s13102-022-00500-7

**Published:** 2022-06-16

**Authors:** Alireza Sadeghi, Mina Rostami, Sahand Ameri, Arezoo Karimi Moghaddam, Zhaleh Karimi Moghaddam, Alireza Zeraatchi

**Affiliations:** 1 Department of Internal Medicine, Vali-E-Asr Hospital, School of Medicine, Zanjan University of Medical Sciences, Zanjan, Iran; 2 Social Determinants of Health Research Center, Zanjan University of Medical Sciences, Zanjan, Iran; 3 Department of Ophthalmology, School of Medicine, Vali-E-Asr Hospital, Zanjan University of Medical sciences, Zanjan, Iran; 4 Department of Radiation Oncology, Vali-E-Asr Hospital, School of Medicine, Zanjan University of Medical Sciences, Zanjan, Iran; 5 Department of Emergency Medicine, Valiasr-E-Asr Hospital, Ayatollah Mousavi Hospital, School of Medicine, Zanjan University of Medical Sciences, Zanjan, Iran

**Keywords:** Isometric exercise, Cervical spondylosis, Neck pain, Disability

## Abstract

**Background:**

Neck pain and disability is a significant public health problem with only very few evidence-based treatment option. The aim of this study was to evaluate the effect of isometric exercise on pain and disability of cervical spondylosis.

**Methods:**

Twenty four patients with cervical osteoarthritis and neck pain (22 females and 2 males; mean age, 46.70 ± 13.71 years) were recruited and randomly allocated into 2 arms: neck isometric exercises (n = 12) and conservative management without exercise (n = 12). The Neck Disability Index (NDI) and Neck Pain and Disability Scale (NPAD) were used to assess participants at baseline and after 4 weeks.

**Results:**

Basic characteristics, NDI score and NPAD score were not significantly different between groups at baseline. The exercise arm demonstrated significantly lower scores regarding NDI (mean, 17.41 vs. 25.58; P-value = 0.035) and NPAD (mean, 25.33 vs. 66.67; P < 0.001), compared to the control arm after 4 weeks. The exercise arm also showed significant within group reduction considering NDI and NPAD scores after 4 weeks (Both, P < 0.001).

**Conclusion:**

Our findings suggested that isometric exercises might be a beneficial treatment for improving pain and disability caused by cervical spondylosis.

*Trial registration* This study was registered at irct.ir (Iranian Registry of Clinical Trials) with the code IRCT20220206053950N1, 07.05.2022, retrospectively registered.

## Background

Being a very common and often debilitating musculoskeletal complaint, neck pain is considered a serious public health problem [1]. According to the statistics, chronic neck pain is responsible for 14.6% of all cases of musculoskeletal problems and annually, 50% of the adult population experience it to some extent [2]. Cervical spondylosis is the most important cause of mechanical neck pain. Also, the most common sites of spondylosis are the joints of the cervical and lumbar vertebrae [4].

Neck pain not only imposes a notable burden on individuals in the society, but also affects families, the healthcare and economic systems of countries. In 2017, age-standardized prevalence, annual incidence, and years lived with disability from neck pain were estimated at 3551, 807, and 352 per 100,000 population worldwide, respectively [3].

Currently, there are several therapeutic approaches, either pain relievers or non-medicinal treatments for the management of cervical spondylosis and its associated pain and disability. Pain medications mainly include non-steroids anti-inflammatory drugs and narcotics with exercise therapy, massage, physiotherapy, and local injections are among the most common non-medicinal therapies. Evidence suggests that exercise therapy plays a role in improving neck pain and disability of patients with cervical spondylosis. Besides, thanks to being non-invasive and profitable, exercise therapy is commonly used in patient rehabilitation [2, 5].

Therapeutic exercises include various workouts such as proprioceptive exercises, stability exercises, strength exercises (dynamic and isometric) and endurance exercises [1, 6].

Isometric exercises (static exercises) strengthen weak muscles without stimulating pain-sensitive structures such as ligaments, tendons, or neck joints, making them more acceptable to the patient. They cause contraction in a specific group of muscles without changing muscle length, impeding involved joints’ movement [7]. Furthermore, ease of use and feasibility make them possible to be done anywhere with no equipment. As for isometric neck exercises which are simple, easy to use and cost-effective, so that may provide patients with a good adherence to the treatment.

Apart from the fact that clinical guidelines suggest therapeutic exercises as an integral part of managing neck pain and disability, prescribing the most advantageous exercise therapy has yet been controversial and even current guidelines do not offer specific recommendations on the preferred type and dosage of exercises [8]. For instance, however, there is some evidence that progressive resistance training of the neck and shoulder muscles may be favorable in reducing neck pain and disability, a recent Cochrane study found that yet there is insufficient evidence to clarify it [8]. Therefore, there seems to be still a need for further studies to evaluate the effect of exercise therapy on improving neck pain and disability so that we decided to design a clinical trial to investigate such effects.

## Methods and material

### Trial design and participants

This was a single-blind randomized clinical trial with a control group (1:1). A total of 42 patients with mild to moderate cervical spondylosis referred to the Rheumatology Clinic of Val-E-Asr Hospital in Zanjan, Iran between January and February 2017 were evaluated for eligibility. The protocol of the present study has been approved by the Research Ethics Committee of Zanjan University of Medical Sciences [ZUMS.REC.1395.222]. Written informed consent was obtained from all participants. This study was conducted in line with Declaration of Helsinki. It should be noted that the control group was also trained exercise programs at the end of the study.

### Group allocation

**Fig. 1:**
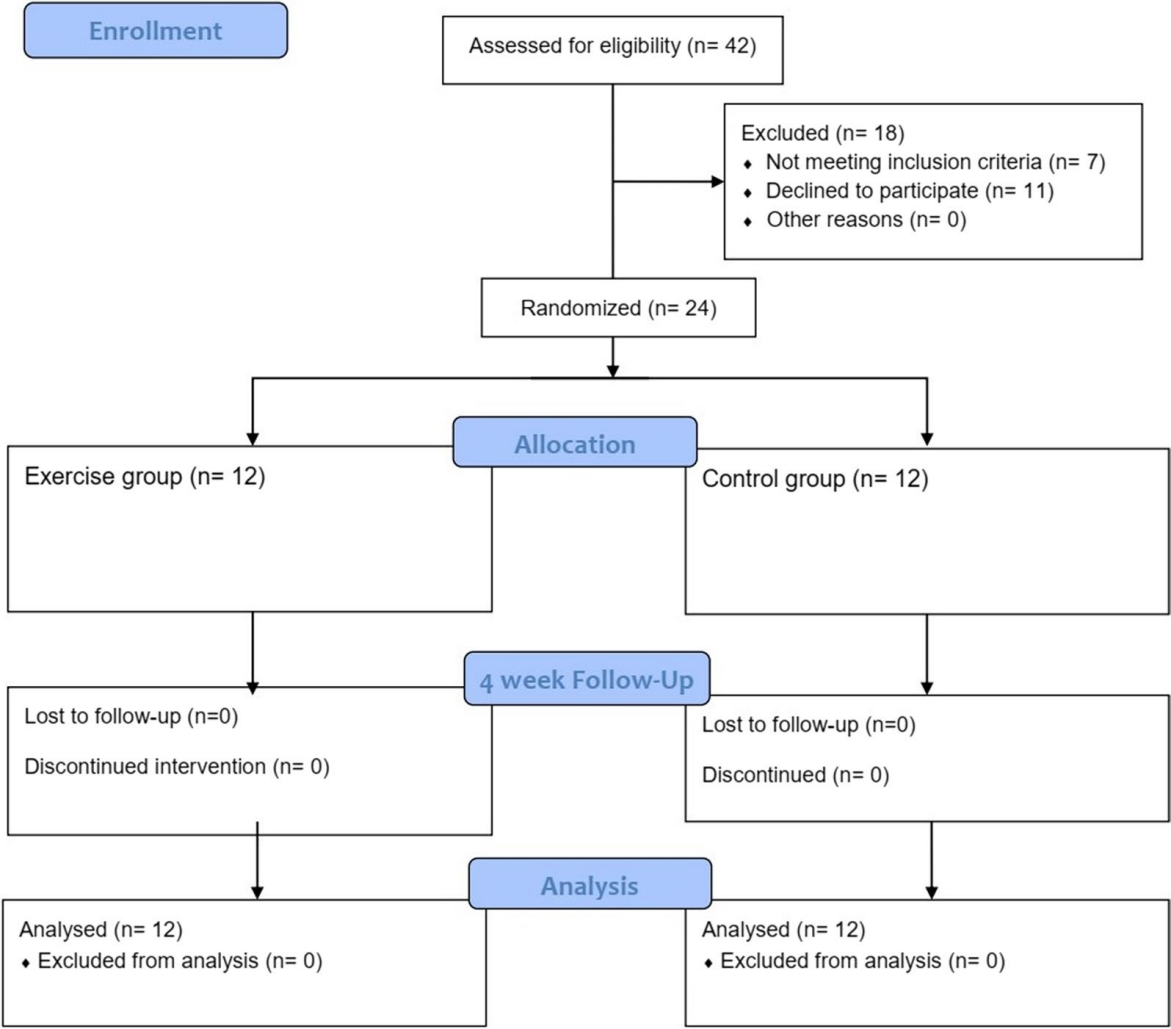
The CONSORT flow diagram

Microsoft Excel software was used to allocate participants randomly to each group using Blocked randomization with randomly varying blocks (block size 4 and 8). Concealed opaque envelopes identifying the assignments to each group were randomly chosen by participants. Data analysts and the outcome assessor were masked. The intervention group received home-based isometric strength exercises and the control group received no intervention. Consolidated Standards of Reporting Trial (CONSORT) diagram is shown in Fig. 1.

### Inclusion and exclusion criteria

Patients over 18 years with clinical findings of cervical spondylosis such as stiffness, chronic neck weakness, radicular or non-radicular neck pain for at least 3 months, without acute cervical nerve root compression, no surgical indications, with a physical examination and signs on cervical magnetic resonance imaging compatible with cervical spondylosis, who had not been receiving exercise therapy or physiotherapy during the 6 weeks prior to the study were included. The diagnosis of cervical spondylosis was made by a board-certified rheumatologist.

We excluded all patients with a history of neck surgery over the past year, a history of inflammatory diseases involving the neck joints, myelopathy, a history of fractures or dislocations of the cervical vertebrae, pregnant women, either patients who did not have a good compliance with the intervention or had difficulty following the study.

### Interventions

Exercise therapy group performed home-based neck isometric strengthening exercises 6 days a week for 4 consecutive weeks as 3 sets/day (morning, afternoon, evening), each set consisted of 6 movements, holding each movement for 10 s, and repeating each 5 times with a 5-s rest between each of them. The control group did not receive the intervention during this period. Exercise programs were taught to patients with details by an experienced physiotherapist at the beginning of the study. In addition, they were provided with handouts clearly explaining the procedure of the exercises. In order to monitor patients’ adherence to the intervention, they were telephoned once a week. Patients in both groups were matched regarding the pain medication consumption and they were asked to take only 500 mg of paracetamol in case of experiencing an unbearable cervical pain.

To do exercises, patients sat in an upright posture. Each set of neck isometric strengthening exercises comprised 6 movements, as follows:

1. Cervical flexion: Lean the neck slightly forward, place palm of both hands on forehead and push the head towards the hands while resisting the movement with hands.
2. Cervical extension: Keep the neck straight, put palm of both hands behind the head, push the head backwards the hands while resisting the movement with hands.
3. Right Lateral Flexion: Keep the neck straight, put palm of right hand on right side of the head, push the head towards the hand to bring head down to the right shoulder while pushing the hand vice versa.
4. Left Lateral Flexion: Keep the neck straight, put palm of left hand on left side of the head, push the head towards the hand to bring head down to the left shoulder while pushing the hand vice versa.
5. Right Rotation: Put palm of right hand on right side of face, rotate the head slightly to the right while resisting the movement with hand.
6. Left Rotation: Put palm of left hand on left side of face, rotate the head slightly to the left while resisting the movement with hand.

### Outcome measures

Neck pain and disability were two main parameters for appraising study outcomes which were measured once at baseline and again 4 weeks later using both Neck Disability Index (NDI) and the Neck Pain and Disability Scale (NPAD). Questionnaires were completed by patients under the supervision of the researcher. The validity and reliability of these questionnaires have already been proved [9]. In Iran, these questionnaires were translated and culturally adapted by Mousavi et al. In 2007 and their validity and reliability were evaluated and introduced as appropriate questionnaires to evaluate the effect of therapeutic interventions on pain and disability caused by neck disorders among Iranian population [10].

### NPAD questionnaire

NPAD is a multi-dimensional questionnaire consisting of 20 items in 4 dimensions of neck problems, pain intensity, effect of neck pain on emotion, and its effect on life activities. Each item is represented by a 10-cm visual analog scale (VAS), on which the patient could mark the severity of pain specific to each item. A score of 0 to 5 has been given to each item; 0 indicates no pain and 5 indicates maximum pain intensity perceived by patients. The total score of the NPAD questionnaire is 100, with lower scores indicating less pain [10]. Cronbach α coefficient of the Persian version of the NPAD sub-scales has been reported to be 0.94, 0.92, 0.84 and 0.75, for neck problems, pain intensity, effect of neck pain on emotion, and its effect on life activities, respectively [10].

### NDI questionnaire

This questionnaire consists of 10 questions, each of which assesses an aspect of disability/pain. These 10 items include pain intensity, personal Care (washing, dressing, etc.), lifting, reading, headaches, concentration, work, driving, sleeping, and recreational activities. The score of each question is calculated from zero to 5. Zero shows no pain/disability and 5 indicates maximum pain/limitation in activity. The total score of the questionnaire is 50 and a higher score indicates a greater disability. For further interpretation, the degree of disability can be categorized according to the score obtained (0–4, no disability; 5–14, mild; 15–24, moderate; 25–34, severe and > 34, complete disability) [10]. The test–retest reliability of the Persian version of the NDI has been shown to be excellent. Cronbach α coefficient of the Persian version of NDI was reported to be 0.88 [10].

### Sample size

G*Power version 3.1 was used to obtain the sample size. The sample size was calculated for both variables of neck pain and disability and the greater sample size was determined based on the study of Hu et al. [11]. For the NDI variable (µ1 = 12.97, µ2 = 17.25, SD1 = 2.98, SD2 = 3.31). With regard to a power of 80%, two-tailed α of 0.05 and a β of 1.35, 11 subjects were calculated per group, which according to the 15% probability of drop-out, at least 12 participants were included in each group.

### Statistical analysis

Data were entered into SPSS software version 18. Descriptive statistics were reported as mean ± standard deviation (SD), if data followed normal distribution, and median (25th, 75th) if data was not distributed normally. Number (%) was used for categorical data. To compare the basic characteristics between the control and intervention groups, Fisher's exact test was performed. For between-group comparison, we used Mann–Whitney U test for NDI and NPAD sub-scales and independent samples t-test for total scores of NDI and NPAD with mean difference (MD) and 95% confidence interval (95%CI). We examined within-group comparison using Wilcoxon signed-rank test for NDI and NPAD sub-scales and paired samples t-test for total scores of NDI and NPAD. For all statistical analysis, a two-tailed alpha level of < 0.05 was considered statistically significant.

## Results

In this study, 24 patients with cervical spondylosis (range, 27 to 50 years) including 22 females (91.7%) and 2 males (8.3%) participated. Each group consisted of 1 male (8.3%) and 11 females (91.7%). The mean ± SD age of total participants was 46.70 ± 13.71 years. With regard to the basic characteristics of the patients, there was no statistically significant difference between two groups (All, *P* > 0.05). (Table 1).

**Table 1:** Basic characteristics of the participants

Variables	Intervention group N (%) / mean ± SD	Control group N (%) / mean ± SD	Total N (%) / mean ± SD	P-value*
Gender				
Male	1 (8.3)	1 (8.3)	2 (8.3)	1.000
Female	11 (91.7)	11 (91.7)	22 (91.7)	
Age, years	49.62 ± 15.43	43.70 ± 11.65	46.70 ± 13.71	0.300
Height	159.00 ± 7.02	161.01 ± 6.45	160.11 ± 6.70	0.458
Weight	64.52 ± 12.20	69.55 ± 10.61	67.02 ± 11.52	0.305
Marital status				
Single	2 (16.7)	0 (0)	2 (8.3)	0.478
Married	10 (83.3)	12 (100)	22 (91.7)	
Education				
HSD >	9 (75.0)	7 (58.3)	16 (66.7)	0.676
HSD	2 (16.7)	3 (25.0)	5 (20.8)	
BS ≤	1 (8.3)	2 (16.7)	3 (12.5)	
Income				
Not sufficient	3 (25.0)	5 (41.7)	8 (33.3)	0.667
Sufficient	9 (75.0)	7 (58.3)	16 (66.7)	
Workload				
Medium	11 (91.7)	9 (75.0)	20 (83.3)	0.590
Heavy	1 (8.3)	3 (25.0)	4 (16.7)	
Type of job				
Mental	2 (16.7)	2 (16.7)	4 (16.7)	0.659
Physical	7 (58.3)	5 (41.6)	12 (50.0)	
Both	3 (25.0)	5 (41.6)	8 (33.3)	
PDH				
Yes	7 (58.3)	5 (41.7)	12 (100)	0.684
No	5 (41.7)	7 (58.3)	12 (100)	

SD, Standard Deviation; HSD, High School Diploma; BS, Bachelor’s Degree; PDH, Past Disease History

*P < 0.05, obtained from Fisher’s Exact test

### NPAD score

The average pre-intervention NPAD total score was 61.67 ± 6.56 and 68.25 ± 23.36 for the intervention and control groups, respectively.

#### Between-group analysis

At the beginning of the study, no statistically significant difference was found between two groups in terms of neither NPAD total score (t (22) = − 0.94, mean difference (MD) = − 6.58; 95% CI, − 21.11, 7.94, *P* = 0.358) nor NPAD sub-scales scores (All, *P* > 0.05).

**Fig. 2:**
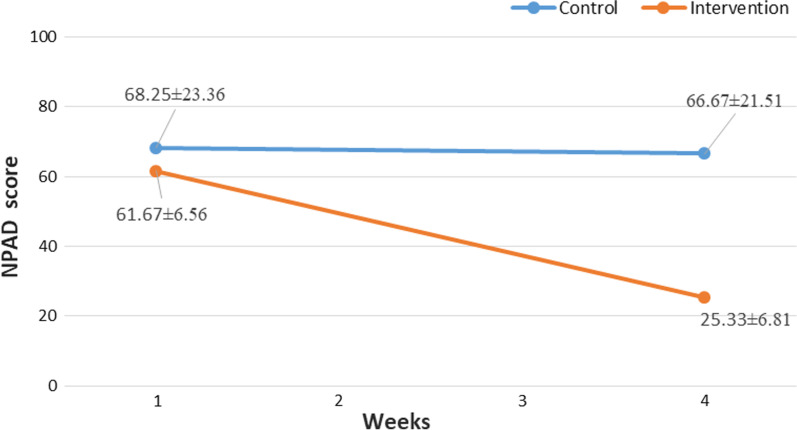
Between-group NPAD scores pre and post-intervention. NPAD, Neck Pain and Disability Scale

After 4 weeks, the patients who received the intervention (Mean = 25.33, SD = 6.81) compared to the patients in the control group (Mean = 66.67, SD = 21.51) reported significantly better total NPAD scores, t(22) = − 6.34, P < 0.001 (Fig. 2). Also, the patients who received the intervention represented significantly lower scores in all sections of the NPAD questionnaire compared to the patients in control group (All, P < 0.05) except for the seventh question (interfering with driving or riding in a car) (*P* = 0.058) (Table 2).

**Table 2:** Between-group NPAD score pre and post-intervention

	Pre-intervention						Post-intervention							
Questions	Intervention group		Control group				Intervention group			Control group				
	Mean Rank	Sum of Ranks	Mean Rank	Sum of Ranks	d	P-value*	Mean Rank	Sum of Ranks		Mean Rank		Sum of Ranks	d	P-value*
How bad is your pain today?	10.29	123.50	14.71	176.50	0.65	0.113	9.50	114.00		15.50		186.00	0.93	**0.030**
How bad is your pain on the average?	12.46	149.50	12.54	150.50	0.01	0.976	6.88	82.50		18.13		217.50	2.68	** < 0.001**
How bad is your pain at its worst?	12.17	146.00	12.83	154.00	0.09	0.781	6.58	79.00		18.42		221.00	3.05	** < 0.001**
Does your pain interfere with your sleep?	10.75	129.00	14.25	171.00	0.51	0.212	8.33	100.00		16.67		200.00	1.45	**0.003**
How bad is your pain with standing?	11.46	137.50	13.54	162.50	0.29	0.461	9.42	113.00		15.58		187.00	0.96	**0.028**
How bad is your pain with walking?	11.92	143.00	13.08	157.00	0.16	0.675	9.67	116.00		15.33		184.00	0.87	**0.040**
Does your pain interfere with driving or riding in a car?	10.42	125.00	14.58	175.00	0.61	0.136	9.83	118.00		15.17		182.00	0.81	0.058
Does your pain interfere with social activities?	10.67	128.00	14.33	172.00	0.53	0.195	8.88	106.50		16.13		193.50	1.19	**0.009**
Does your pain interfere with recreational activities?	10.04	120.50	14.96	179.50	0.75	0.078	8.33	100.00		16.67		200.00	1.45	**0.003**
Does your pain interfere with work activities?	12.21	146.50	12.79	153.50	0.08	0.824	6.67	80.00		18.33		220.00	2.91	** < 0.001**
Does your pain interfere with your personal care (eating, dressing, bathing, etc.)?	12.00	144.00	13.00	156.00	0.14	0.718	9.00	108.00		16.00		192.00	1.13	**0.013**
Does your pain interfere with your personal relationship (family, friends, sex, etc.)?	13.08	157.00	11.92	143.00	0.16	0.677	7.88	94.50		17.13		205.50	1.72	**0.001**
How does your pain change your outlook on life and future (depression, hopelessness)?	11.46	137.50	13.54	162.50	0.29	0.457	7.54	90.50		17.46		209.50	1.96	** < 0.001**
Does your pain affect your emotions?	12.04	144.50	12.96	155.50	0.13	0.741	8.42	101.00		16.58		199.00	1.41	**0.004**
Does your pain affect your ability to think or concentrate?	14.08	169.00	10.92	131.00	0.46	0.252	8.75	105.00		16.25		195.00	1.25	**0.008**
How stiff is your neck?	12.46	149.50	12.54	150.50	0.01	0.976	8.50	102.00		16.50		198.00	1.37	**0.005**
How much trouble do you have turning your neck?	13.63	163.50	11.38	136.50	0.32	0.414	7.63	91.50		17.38		208.50	1.90	**0.001**
How much trouble do you have turning your neck (look up and down)?	13.08	157.00	11.92	143.00	0.16	0.657	7.88	94.50		17.13		205.50	1.72	**0.001**
How much trouble do you have working overhead?	11.67	140.00	13.33	160.00	0.23	0.545	8.54	102.50		16.46		197.50	1.35	**0.005**
How much do pain pills help?	12.63	151.50	12.38	148.50	0.03	0.930	8.54	102.50		16.46		197.50	1.35	**0.005**
Total NPAD score, MD (95% CI)	61.67 ± 6.56	68.25 ± 23.36	-6.58 (-21.11, 7.94)		-0.38	0.358	25.33 ± 6.81		66.67 ± 21.51		-41.33 (-54.84, -27.82)		-2.58	** < 0.001**

*P < 0.05, All P-values are obtained from Mann–Whitney U test except for total NPAD score obtained from independent samples t-test

Significant P-values are showed in bold

MD, Mean Difference, NPAD, Neck Pain and Disability, d, Effect size (Cohen’s d)

#### Within-group analysis

Regarding the intervention group, the results from the pre-intervention (Mean = 61.67, SD = 6.56) and post-intervention (Mean = 25.33, SD = 6.81) showed that receiving exercise therapy resulted in an improvement in NPAD total score, t(11) = − 11.58, *P* < 0.001. In accretion, all NPAD sub-scale scores demonstrated a significant reduction among the patients in the intervention group (All, *P* < 0.05).

In contrast, within-group analysis of the control group revealed no statistically significant improvement considering NPAD total score (68.25 ± 23.36 Vs. 67.08 ± 21.37, t(11) = 0.86, *P* = 0.405). (Table 3).

**Table 3:** Within-group NPAD score comparison

	Intervention group							Control group							
Questions	Pre-intervention		Post-intervention					Pre-intervention			Post-intervention				
	Mean Rank	Sum of Ranks	Mean Rank		Sum of Ranks	d	P-value*	Mean Rank	Sum of Ranks		Mean Rank		Sum of Ranks	d	P-value*
How bad is your pain today?	6.00	66.00	0		0	1.56	**0.003**	7.00	28.00		6.25		50.00	0.52	0.380
How bad is your pain on the average?	6.00	66.00	0		0	1.50	**0.003**	0	0		0		0	0	1.000
How bad is your pain at its worst?	6.50	78.00	0		0	1.60	**0.002**	1.50	3.00		0		0	0.89	0.157
Does your pain interfere with your sleep?	5.00	45.00	0		0	1.31	**0.007**	2.00	2.00		1.00		1.00	0.26	0.655
How bad is your pain with standing?	5.00	45.00	0		0	1.31	**0.007**	0	0		0		0	0	1.000
How bad is your pain with walking?	4.42	26.50	1.50		1.50	0.96	**0.034**	0	0		0		0	0	1.000
Does your pain interfere with driving or riding in a car?	6.15	61.50	4.50		4.50	1.28	**0.008**	2.50	2.50		2.50		7.50	0.60	0.317
Does your pain interfere with social activities?	5.44	43.50	1.50		1.50	1.18	**0.012**	0	0		1.00		1.00	0.60	0.317
Does your pain interfere with recreational activities?	4.50	36.00	0		0	1.23	**0.010**	0	0		0		0	0	1.000
Does your pain interfere with work activities?	6.50	78.00	0		0	1.62	**0.002**	0	0		1.00		1.00	0.60	0.317
Does your pain interfere with your personal care (eating, dressing, bathing, etc.)?	6.05	60.50	5.50		5.50	1.16	**0.014**	0	0		1.00		1.00	0.60	0.317
Does your pain interfere with your personal relationship (family, friends, sex, etc.)?	5.94	53.50	1.50		1.50	1.29	**0.008**	0	0		1.50		3.00	0.89	0.157
How does your pain change your outlook on life and future (depression, hopelessness)?	5.50	55.00	0		0	1.40	**0.005**	3.00	3.00		1.50		3.00	0	1.000
Does your pain affect your emotions?	3.50	21.00	0		0	1.00	**0.027**	2.25	4.50		1.50		1.50	0.48	0.414
Does your pain affect your ability to think or concentrate?	6.50	78.00	0		0	1.60	**0.002**	2.50	2.50		2.50		7.50	0.60	0.317
How stiff is your neck?	6.00	66.00	0		0	1.54	**0.003**	1.00	1.00		0		0	0.60	0.317
How much trouble do you have turning your neck?	6.00	66.00	0		0	1.51	**0.003**	2.25	4.50		1.50		1.50	0.48	0.414
How much trouble do you have turning your neck (look up and down)?	6.50	78.00	0		0	1.61	**0.002**	1.50	3.00		0		0	0.89	0.157
How much trouble do you have working overhead?	6.00	60.00	6.00		6.00	1.14	**0.015**	4.50	18.00		6.86		48.00	0.83	0.178
How much do pain pills help?	6.50	65.00	6.50		13.00	0.92	**0.040**	3.50	10.50		3.50		10.50	0	1.000
Total NPAD score, MD (95% CI)	61.67 ± 6.56	25.33 ± 6.814		-36.33 (-43.23, -29.43)		-3.34	** < 0.001**	68.25 ± 23.36		67.08 ± 21.37		1.16 (-1.79, 4.13)		0.047	0.405

^*^P < 0.05, All P-values are obtained from Wilcoxon signed-rank test except for total NPAD score obtained from paired samples t-test

Significant P-values are showed in bold

MD, Mean Difference, APAD, Neck Pain and Disability, d, Effect size (Cohen’s d)

### NDI score

The mean pre-intervention NDI total score was 27.08 ± 9.78 among patients of the intervention group and 25.41 ± 10.78 for patients of the control group.

#### Between-group analysis

In terms of pre-intervention NDI total score, an independent samples t-test revealed no statistically significant difference between two groups, t (22) = 0.39, MD = 1.66; 95% CI, − 7.04, 10.38, *P* = 0.696. Furthermore, none of the pre-intervention NDI sub-scales scores were significantly different between two groups (All, P > 0.05). (Table 4).

**Table 4:** Between-group NDI score pre and post-intervention

	Pre-intervention						Post-intervention					
Sections	Intervention group		Control group				Intervention group		Control group			
	Mean Rank	Sum of Ranks	Mean Rank	Sum of Ranks	d	P-value*	Mean Rank	Sum of Ranks	Mean Rank	Sum of Ranks	D	P-value*
Pain intensity	12.67	152.00	12.33	148.00	0	1.000	9.13	109.50	15.88	190.50	1.086	**0.017**
Personal care (washing, dressing, etc.)	13.29	159.50	11.71	140.50	0	1.000	11.33	136.00	13.67	164.00	0.335	0.443
Lifting	12.63	151.50	12.38	148.50	0.570	0.180	9.79	117.50	15.21	182.50	0.829	0.060
Reading	13.04	156.50	11.96	143.50	0.417	0.317	10.25	123.00	14.75	177.00	0.671	0.128
Headaches	11.25	135.00	13.75	165.00	0.756	0.083	8.33	100.00	16.67	200.00	1.459	**0.003**
Concentration	13.33	160.00	11.67	140.00	0.237	0.564	10.63	127.50	14.38	172.50	0.550	0.198
Work	14.13	169.50	10.88	130.50	0.417	0.317	11.00	132.00	14.00	168.00	0.434	0.319
Driving	13.00	156.00	12.00	144.00	0.417	0.317	9.13	109.50	15.88	190.50	1.086	**0.017**
Sleeping	11.58	139.00	13.42	161.00	0.603	0.157	10.67	128.00	14.33	172.00	0.537	0.219
Recreation	14.50	174.00	10.50	126.00	0.603	0.157	13.00	156.00	12.00	144.00	0.142	0.755
Total NDI score, MD (95% CI)	27.08 ± 9.78	25.41 ± 10.78	1.66 (-7.04, 10.38)		0.162	0.696	17.41 ± 7.10	25.58 ± 10.38	-8.16 (− 15.70, − 0.63)	− 0.918	0.035	**0.035**

*P < 0.05, All P-values are obtained from Mann–Whitney U test except for total NDI score obtained from independent samples t-test

Significant P-values are showed in bold

MD, Mean Difference, NDI, Neck Disability Index, d, Effect size (Cohen’s d)

**Fig. 3:**
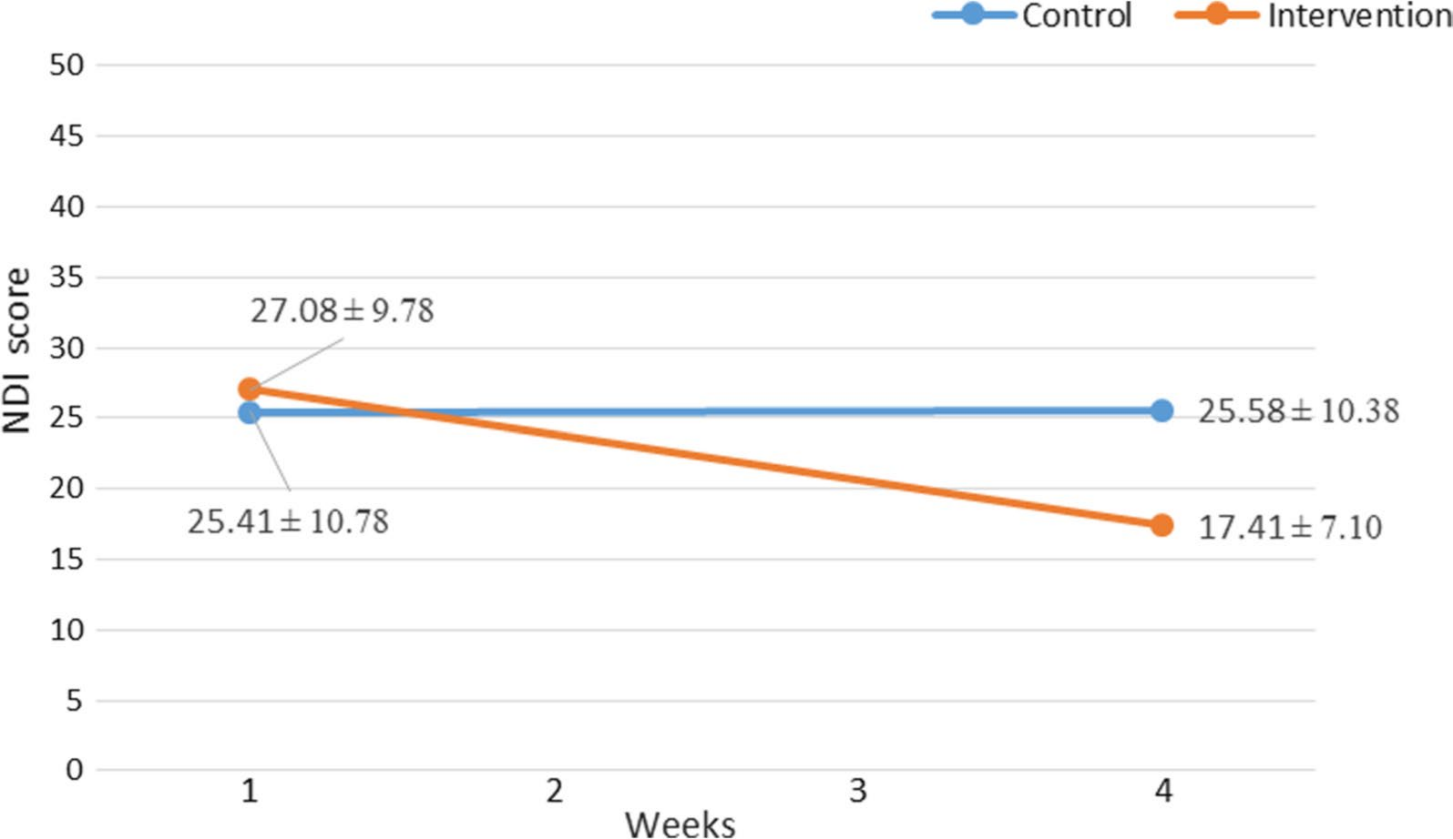
Between-group NDI scores pre and post-intervention. NDI, Neck Disability Index

After 4 weeks, patients of intervention group reported statistically significantly lower scores in comparison with control group (17.41 ± 7.10 Vs. 25.58 ± 10.38, t (22) = − 2.24, MD = − 8.16; 95% CI, − 15.70, − 0.63, *P* = 0.035). Moreover, a Mann–Whitney test uncovered that pain Intensity (Median (Mdn), 1.00 vs. 3.00, U = 31.50, Z = − 2.43, *P* = 0.017), headaches (Mdn, 1.50 vs. 3.00, U = 22.00, Z = − 3.00, *P* = 0.003), and driving (Mdn, 2.00 vs. 3.00, U = 31.50, Z = − 2.47, *P* = 0.017) sub-scales scores of patients in intervention group were statistically significantly lower than those in patients of control group with large effect sizes (pain Intensity, d = 1.08, headaches, d = 1.45, driving, d = 1.08). (Table 4) (Fig. 3).

#### Within-group analysis

Patients in the intervention group reported significantly lower NDI total scores after 4 weeks of receiving exercise therapy (Mean = 27.08, SD = 9.78) compared to the pre-intervention scores (Mean = 17.41, SD = 7.10), *t*(11) = 6.58, *p* < 0.001). Nevertheless, considering the control group, patients showed no improvement in NDI total score after 4 weeks, (Mean ± SD, 25.41 ± 10.78 vs. 25.58 ± 10.38, *t*(11) = − 0.35, *P* = 0.732).

A Wilcoxon signed-rank test indicated that exercise therapy resulted in a statistically significant decrease in all NDI sub-scales scores among patients in the intervention group after 4 weeks (All, *P* < 0.05), whereas, patients in the control group indicated no significant decline in terms of all NDI sub-scales scores (All, *P* > 0.05). (Table 5).

**Table 5:** Within-group NDI score comparison*

	Intervention group						Control group					
Sections*	Pre		Post				Pre		Post			
	Mean Rank	Sum of Ranks	Mean Rank	Sum of Ranks	d	P-value	Mean Rank	Sum of Ranks	Mean Rank	Sum of Ranks	d	P-value
Pain intensity	0	0	6.50	78.00	1.79	**0.001**	1.50	1.50	1.50	1.50	0	1.000
Personal care (washing, dressing, etc.)	0	0	4.00	28.00	1.16	**0.014**	0	0	0	0	0	1.000
Lifting	0	0	3.50	21.00	1.01	**0.027**	1.50	3.00	0	0	0.570	0.180
Reading	2.50	2.50	4.79	33.50	1.00	**0.028**	1.00	1.00	0	0	0.417	0.317
Headaches	0	0	5.00	45.00	1.35	**0.006**	0	0	2.00	6.00	0.756	0.083
Concentration	0	0	4.50	36.00	1.22	**0.01**	2.00	4.00	2.00	2.00	0.237	0.564
Work	0	0	5.00	45.00	1.33	**0.006**	1.00	1.00	0	0	0.417	0.317
Driving	0	0	5.00	45.00	1.33	**0.007**	1.00	1.00	0	0	0.417	0.317
Sleeping	0	0	4.00	28.00	1.28	**0.008**	0	0	1.50	3.00	0.603	0.157
Recreation	0	0	3.50	21.00	1.15	**0.014**	1.50	3.00	0	0	0.603	0.157
Total NDI score, MD (95% CI)	27.08 ± 9.78	17.41 ± 7.10	− 9.66 (− 6.43, − 12.89)		− 3.66	** < 0.001**	25.41 ± 10.78	25.58 ± 10.38	0.16 (− 1.21, 0.87)		0.015	0.732

^*^ P < 0.05, All P-values are obtained from Wilcoxon signed-rank test except for total NPAD score obtained from paired samples t-test

Significant P-values are showed in bold

MD, Mean Difference, NDI, Neck Disability Index, d, Effect size (Cohen’s d)

NDI total scores were interpreted (no disability, 0–4; mild, 5–14; moderate, 15–24; severe, 25–34; complete, above 34). As can be seen in Table 6, however, at baseline, 8 patients (66.7%) of the intervention group were in severe disability category and one (8.3%) was in complete disability category, After 4 weeks, only one patient developed severe disability and no one experienced a complete disability.

**Table 6:** NDI total score interpretations by Intervention and control groups pre and post-intervention

	Pre-intervention			Post-intervention		
Disability*	Intervention group N (%)	Control group N (%)	Total N (%)	Intervention group N (%)	Control group N (%)	Total N (%)
No disability	1.0 (8.3)	0 (0)	1.0 (4.2)	1.0 (8.3)	0 (0)	1.0 (4.1)
Mild	0 (0)	2.0 (16.7)	2.0 (8.3)	2.0 (16.7)	2.0 (16.7)	4.0 (16.7)
Moderate	2.0 (16.7)	4.0 (33.3)	6.0 (25.0)	8.0 (66.7)	4.0 (33.3)	12.0 (50.0)
Severe	8.0 (66.7)	3.0 (25.0)	11.0 (45.8)	1.0 (8.3)	3.0 (25.0)	4.0 (16.7)
Complete	1.0 (8.3)	3.0 (25.0)	4.0 (16.7)	0 (0)	3.0 (25.0)	3.0 (12.7)

*No disability, 0–4; mild, 5–14; moderate, 15–24; severe, 25–34; complete, above 34

## Discussion

We revealed that performing neck isometric strength exercises for 4 consecutive weeks significantly alleviated neck pain and disability among patients suffering from chronic neck pain.

We conducted a randomized trial with control group receiving no therapeutic exercise. In a Cochrane review study, Gross et al. [7] stated that inclusion of trials comparing a single exercise intervention with either a control group (No exercise therapy) or a comparative group (Exercise plus another intervention) might optimize assessing the therapeutic effect of exercise interventions. As there are numerous studies in which the intervention group/groups were compared with the control group, undergoing either health promotion activities or no exercise [12–18].

Since neck pain and disability tend to be recurrent in patients, it is generally accepted that the effectiveness of exercise therapy as a therapeutic approach should be considered a top-priority for researchers to investigate [19].

It has been shown that in patients with chronic neck pain, deep neck flexors and extensors atrophy and altered electromyography activity is evident. In other words, it is believed that these structural and functional alterations of deep cervical muscles are a reason for chronic and recurrent neck pain [20]. These group of muscles can gain strength thanks to isometric exercises.

A recent study has demonstrated that isometric neck exercises failed to significantly enhance neck strength of elite women's football-code athletes after a 12-week follow-up period in comparison to the control group [21]. In another study, as evidenced by Sowmya, neck pain and disability significantly improved after 3 weeks among both intervention groups (dynamic and isometric neck exercises) compared with the control group, however, dynamic neck exercises were found to be much more beneficial in this regard [22].

The majority of studies indicate a significant effect of isometric neck exercises in reducing neck pain and disability. Nevertheless, there is still a lack of evidence to propose the optimal dosage in order to achieve a clinical efficacy.

Gupta et al. compared the effectiveness of deep cervical flexor (DCF) training with conventional neck isometric training (CIT) among 30 patients with chronic neck pain. They revealed that after four weeks However, DCF was more significantly effective in comparison to CIT, in within-group analysis both exercise therapies were significantly beneficial for reducing neck pain and disability. Similarly, in our study patients also were followed for 4 weeks and we both used NDI to assess disability, while Gupta et al. measured the neck pain using VAS score in spite of our study which NPAD was used for this purpose. Our results is in line with the aforementioned study, as we both found the CIT to play a significant role in relieving neck pain and disability among patients with chronic neck pain [20].

In a randomized controlled trial (RCT) of 30 patients with non-specific neck pain that is in line with the findings of our study, Shoukat et al. reported that although, after a 6-week follow-up duration, multiple-angle neck isometrics were significantly more favorable in improving neck pain and disability than isometric neck exercises in neutral spine, both interventions decreased significantly neck pain and disability. They used VAS and NDI to evaluate study outcomes and had a slightly longer follow-up period than ours [23].

The effectiveness of velocity‐specific exercise program and isometric exercise program were examined in a 6-week follow-up RCT. The authors of said study found that there was no statistically significant difference between two interventions, both resulting in a considerable improvement in terms of cervical muscles function and performance [24].

Khan et al. compared the effects of isometric neck exercises with general neck exercises in a 12-week RCT by applying VAS and north wick Park neck pain questionnaire to assess neck pain and disability in patients with chronic non-specific neck pain. Even though, they demonstrated that either intervention had a significant impact on reducing neck pain and disability, Isometric exercises reported to be clinically more beneficial than general exercises [25].

In most studies that outcomes have been measured at different time points, a significant impact of exercise therapy has been shown at the end of the shortest duration (i.e. 4 weeks) along with the further time points (i.e. 6, 8 weeks) as has been shown in Chung et al. and Li et al. studies [26, 27]. In fact, this observation implies that therapeutic exercises may be significantly effective in a short duration as we also revealed in the present study.

Chung et al. in a study to assess the effectiveness of Cranio-cervical flexion exercise in comparison with neck isometric exercise in patients with chronic neck pain, found that both interventions significantly improved pain (VAS score) and perceived disability (NDI score) in patients, after 4 and 8 weeks of undergoing exercisers and there were not any significant differences between two groups considering neck pain and disability [26].

In Li’s study women with chronic neck pain were allocated into three groups including, progressive resistance training (PRT), fixed resistance training (FRT), and control group (No intervention). The outcomes of neck pain and disability were measured using VAS and NDI. They reported that both intervention groups (PRT, FRT) were significantly superior to the control group at either 4 or 6 weeks of receiving therapeutic exercises [27].

Most studies have evaluated the effectiveness of exercise therapy by means of measuring neck pain and disability using VAS and NDI scores. However, in the present study, the NPAD was used to examine neck pain, which covers various aspects of the patients’ pain, so that in spite of the VAS score, is not limited to the patient's perceived pain in its general sense.

## Limitations

This study has some limitations. We considered only one end-point time (4 weeks) to follow up patients instead of various time-points (6, 8 weeks or 1 year) to measure study outcomes. Another limitation of the study, may be relying on the self-report questionnaires to measure study outcomes only, which may be a potential source of bias in the study. For instance, considering methods of measuring the strength, function and active range of motion of the muscles in addition to utilizing self-report questionnaires seems more reliable.

## Conclusion

Together, the results of the present study showed that isometric neck exercises had a significant impact on reducing cervical pain and disability among patients with cervical spondylosis, within 4 weeks of receiving the exercises.

## Data Availability

The datasets used and analyzed during the current study are available from the corresponding author on reasonable request.

## Abbreviations

NDI: Neck Disability Index
NPAD: Neck Pain and Disability Scale
PRT: Progressive Resistance Training
SD: Standard Deviation
MD: Mean Difference
Mdn: Median
RCT: Randomized Controlled Trial
FRT: Fixed Resistance Training
DCF: Deep Cervical Flexor
CIT: Conventional Isometric Training
